# Supplementary material for: Benefits of realist evaluation for rapidly changing health service delivery
Source: BMJ Open. 2022 Jul 27;12(7):e060347. doi: 10.1136/bmjopen-2021-060347 (PMC9335061; doi:10.1136/bmjopen-2021-060347)
Supplement: Author's manuscript [file bmjopen-2021-060347.draft_revisions.pdf]

BMJ Open is committed to open peer review. As part of this commitment we make the peer review history of every article we publish publicly available.

When an article is published we post the peer reviewers' comments and the authors' responses online. We also post the versions of the paper that were used during peer review. These are the versions that the peer review comments apply to.

The versions of the paper that follow are the versions that were submitted during the peer review process. They are not the versions of record or the final published versions. They should not be cited or distributed as the published version of this manuscript.

BMJ Open is an open access journal and the full, final, typeset and author-corrected version of record of the manuscript is available on our site with no access controls, subscription charges or pay-per-view fees (http://bmjopen.bmj.com).

If you have any questions on BMJ Open's open peer review process please email info.bmjopen@bmj.com

BMJ Open

Using Realist Evaluation in the Study of Rapidly Evolving Service Delivery Models: Reflections from the FRONTIER study on First-Contact Physiotherapy

Journal:	*BMJ Open*
Manuscript ID	bmjopen-2021-060347.R1
Article Type:	Communication
Date Submitted by the Author:	05-Feb-2022
Complete List of Authors:	Jagosh, Justin; University of the West of England<br>Stott, Hannah; University of the West of England<br>Halls, Serena; University of the West of England<br>Thomas, Rachel; University of the West of England<br>Liddiard, Cathy; University of the West of England<br>Cupples, Margaret; Queens University, Belfast, General Practice<br>Cramp, Fiona; The University of the West of England, Allied health professionals<br>Kersten, Paula; Canterbury Christ Church University, School of Health Sciences<br>Foster, Dave; University of the West of England<br>Walsh, Nicola; University of the West of England, Faculty of Health and Applied Sciences, Glenside Campus
**Primary Subject Heading**:	General practice / Family practice
Secondary Subject Heading:	General practice / Family practice
Keywords:	COVID-19, EDUCATION & TRAINING (see Medical Education & Training), PRIMARY CARE

SCHOLARONE™
Manuscripts

I, the Submitting Author has the right to grant and does grant on behalf of all authors of the Work (as defined in the below author licence), an exclusive licence and/or a non-exclusive licence for contributions from authors who are: i) UK Crown employees; ii) where BMJ has agreed a CC-BY licence shall apply, and/or iii) in accordance with the terms applicable for US Federal Government officers or employees acting as part of their official duties; on a worldwide, perpetual, irrevocable, royalty-free basis to BMJ Publishing Group Ltd ("BMJ") its licensees and where the relevant Journal is co-owned by BMJ to the co-owners of the Journal, to publish the Work in BMJ Open and any other BMJ products and to exploit all rights, as set out in our licence.

The Submitting Author accepts and understands that any supply made under these terms is made by BMJ to the Submitting Author unless you are acting as an employee on behalf of your employer or a postgraduate student of an affiliated institution which is paying any applicable article publishing charge ("APC") for Open Access articles. Where the Submitting Author wishes to make the Work available on an Open Access basis (and intends to pay the relevant APC), the terms of reuse of such Open Access shall be governed by a Creative Commons licence – details of these licences and which Creative Commons licence will apply to this Work are set out in our licence referred to above.

Other than as permitted in any relevant BMJ Author's Self Archiving Policies, I confirm this Work has not been accepted for publication elsewhere, is not being considered for publication elsewhere and does not duplicate material already published. I confirm all authors consent to publication of this Work and authorise the granting of this licence.

For peer review only

Using Realist Evaluation in the Study of Rapidly Evolving Service Delivery Models: Reflections from the FRONTIER study on First-Contact Physiotherapy

Justin Jagosh (Corresponding Author)
Centre for Advancement in Realist Evaluation and Synthesis (CARES)
justin.jagosh@realistmethodology-cares.org

Hannah Stott
University of the West of England

Serena Halls
University of the West of England

Rachel Thomas
University of the West of England

Cathy Liddiard
University of the West of England

Margaret Cupples
Queen's University, Belfast

Fiona Cramp
University of the West of England

Paula Kersten
Canterbury Christ Church University

Dave Foster
Patient Research Partner

Nicola Walsh
University of the West of England

For peer review only

Abstract: Realist Evaluation is a methodology that addresses the questions, 'what works, for whom, in which circumstances, and how?' In times of rapid context change, the approach may be advantageous in relation to counter-factual statistical investigations such as randomized controlled trials, as the latter may require relatively stable implementation processes and contexts to fairly assess comparisons. With realist evaluation, researchers cycle through iterative phases of theory development testing for interventions if they are evolving in real time. Reflecting on our work conducting a realist evaluation of First-Contact Physiotherapy (FCP) during the COVID-19 pandemic, we identify five important considerations for assessing rapidly evolving service delivery models. These include to: (1) ensure initial programme theories are formulated via creative thinking sessions, literature, and stakeholder consultation; (2) test the causal impact of formal and informal (e.g., emergent) components of service delivery models. (3) theorize using comparisons and rival theory statements; (4) envision broad system impacts beyond the immediate implementation setting; and (5) incorporate rapidly evolving service developments and context changes (e.g COVID-19) into the theory testing process in real-time. Through the reflections presented in this paper, we hope our demonstration of realist evaluation and the FRONTIER experience will help other teams improve the design of studies used to assess emerging and rapidly changing service delivery models.

For peer review only

For peer review only - http://bmjopen.bmj.com/site/about/guidelines.xhtml

Introduction

Realist methodology is used for investigating how programmes work, for whom and in which circumstances [1, 2]. Although gaining widespread recognition for its value in assessing complexity across health and social service sectors [3], guidance is sparse on using the realist approach to assess rapidly evolving models of health service delivery. Health services can evolve due to shifting clinical need, competing health policies, and resource scarcity. Initiatives may also evolve due to practical necessities realized only during the service embedding process. Complicating the evaluation of such new efforts is the layering of interventions on existing services to address deficits and meet the needs of increasing demand. As trends and political priorities shift, new service delivery models may be terminated before substantial evidence of success or failure can accumulate. The resources of terminated interventions are often re-purposed for newer initiatives all within the reality of ever-evolving contexts. Realist evaluation is a suitable methodology to assess interventions in times of rapid change as it brings attention to key mechanisms of programmes and aspects of the context that matter, and such insights can be taken forward to other studies as programmes evolve and are re-purposed. In this way, realist evaluations cumulate so as to not “re-invent the wheel” with every study and learn from the relevant programme theorizing that has come before [1].

Understanding Realist Evaluation: Realist evaluations of service delivery models typically involve the development of initial programme theories (IPTs) which are causal statements (e.g., ‘if…then’) hypothesizing how programme strategies and components work to produce

For peer review only - http://bmjopen.bmj.com/site/about/guidelines.xhtml

outcomes. Protocols are developed to collect and analyse data to test the IPTs. Deductive (theory-testing) as well as inductive (theory-gleaning) approaches are used to build a retroductive analysis to uncover underpinning explanatory mechanisms [4, 5]. Realist evaluation uses context-mechanism-outcome (CMO) configurations to achieve generative explanatory insights. Programme mechanisms are underpinning generative forces in terms of how people respond to resources [6]. Context may include elements of causal impact outside the set of formal programme resources [2, 7, 8]. Outcomes are typically understood as measurable impacts at the behavioural, clinical or systems level. Realist evaluations are sometimes accompanied by a realist review [9], which is a literature-based analysis of programme theories related to the intervention under scrutiny.

In this paper, we reflect on the FRONTIER study, which is a UK-based *National Institute for Health Research* (NIHR) funded study https://fundingawards.nihr.ac.uk/award/16/116/03 examining First-Contact Practitioner (Physiotherapist) (FCPP). FCPP introduces specialist and advanced practice physiotherapy within primary care to assess, diagnose, treat and discharge patients presenting with musculoskeletal (MSK) disorders without the requirement for a prior general practitioner (GP) consultation [10]. FCPP is a model developed in contrast to many traditional service delivery models for MSK management in which patients are seen by a physiotherapist only after receiving a referral from a GP or consultant. FCPPs are intended to work in directly in the primary care setting, allowing patients to self-refer or book via reception staff who have added decision-making responsibilities for the care pathway for MSK patients. Our realist evaluation has revealed that the FCPP embedding process is complex and impacts GPs, reception staff, patients, physiotherapists, and the wider health system. We discuss five

For peer review only - http://bmjopen.bmj.com/site/about/guidelines.xhtml

important considerations for assessing rapidly evolving service delivery models using the realist evaluation methodology.

Table 1: Five Points of Guidance on Conducting a Realist Evaluation of Rapidly Evolving Service Delivery

1. Develop initial programme theories using team-based brainstorming, literature, and stakeholder consultation.<br><br>2. Test causal impact of both the formal and informal (e.g., emergent) architecture of service delivery models.<br><br>3. Theorize using comparisons and rival theory statements.<br><br>4. Envision broad system impacts beyond the immediate implementation settings.<br><br>5. Incorporate rapidly evolving service developments and context changes (e.g COVID-19) into the theory testing process in real-time.

For peer review only

1. Develop initial programme theories using a combination of team-based brainstorming, literature, and stakeholder consultation.

Published high-quality literature is often sought to inform the development of IPTs at the outset of a realist evaluation. However new and progressed service delivery models lack a historical trail of evidence regarding success and failure, nor of details on the programme's architecture, especially the informal aspects. These unknowns preclude easy identification of IPTs from pre-existing literature. For this reason, IPT development requires creative thinking and consultation with key stakeholders at the early stages. Such activities will bring IPT theorizing to the current programme developments, and likely produce hypothetical insights

For peer review only - http://bmjopen.bmj.com/site/about/guidelines.xhtml

about the mechanisms at play, which may be obscured in literature sources [11, 12]. Data sources can be expanded to include unpublished (grey) literature, policy documents, online blogs and forums, and professional body documentation.

Lessons from FRONTIER: For the FRONTIER project, a six-month realist synthesis was scheduled at the outset of the project to develop the IPTs and establish a suitable research scope to examine the most important facets of the architecture of the emerging FCPP model. The body of high-quality empirical literature on FCPP was found to be limited and papers did not reveal clearly articulated programme theories that could be imported to the current study. However, the literature was helpful to stimulate creative thinking and discussion within the team, which informed the development of the IPTs. We found that online blogs in which physiotherapists candidly talked about their experience of working in general practice provided greater insight into contemporary implementation issues than the published empirical work.

2. Test causal impact of both the formal and informal (e.g. emergent) architecture of service delivery models.

Programme architecture constitutes the spectrum of strategies and resources that are both formally allocated through policy guidance as well as assembled and adapted informally from existing resources in the context of implementation. IPTs developed and tested in a realist evaluation can account for both the formal and informal architecture of programmes. Policy documentation on service re-organization often involves descriptions of formal architecture. However, implementation processes require, for example, local-level skills such as communication and collaborative know-how. The theorizing process in a realist evaluation can

For peer review only - http://bmjopen.bmj.com/site/about/guidelines.xhtml

begin with a consideration of formal strategies, followed by informal strategies explaining the success of embedding programmes over time. The combination of formal and informal theories may form a complete picture of how the programme evolves in real time.

Lessons from FRONTIER:

Taking FCPP banding (i.e., payscale and associated skill-level) as an example, documents from Health Education England (HEE) were scrutinized to understand the recommendations for how FCPP roles should be implemented in primary care settings. HEE states that "a First Contact Practitioner (FCP) is a diagnostic clinician working in Primary Care at the top of their clinical scope of practice at masters level Agenda for Change Band 7 or equivalent and above. This allows the FCP to be able to assess and manage undifferentiated and undiagnosed MSK presentations" [13]. However, banding for FCPP roles vary. We determined that the architecture of FCPP changes with banding such that those physiotherapists who have greater experience and clinical capability and therefore may be able to prescribe and inject are potentially vulnerable to time pressures in a primary care clinic, whereas those physiotherapists, who cannot prescribe and inject, use traditional physiotherapy approaches such as physical exercise, education and communication with the patient (in opposition to injections and prescription). Paradoxically, lower banded physiotherapists may provide traditional forms of physiotherapy with a required appointment length of 20 minutes, whereas higher banded professionals may lean on injections and prescriptions thus reducing appointment length. These differences may impact clinical and cost outcomes in unexpected ways. While formal guidance outlines general principles of FCP banding and pay structure,

For peer review only - http://bmjopen.bmj.com/site/about/guidelines.xhtml

variation in banding at the local level means that primary care managers are left on their own to try to understand and make decisions regarding FCPP service architecture.

3. Theorize using comparisons and rival theory statements

Initial programme theories that contrast new initiatives with older established practices can be important to determine clinical and cost effectiveness of new efforts. Contrastive statements in the development of IPTs need to include 'how, why, and for whom' content to fit with the retroductive theorizing objective of realist evaluation. This is different from counter-factual causal analysis [14] in which causal claims are established through high-volume correlation (A leads to B across many cases). Two advantages in including realist contrastive and rival theories are (a) to help determine if new initiatives layered on existing services yield at least no worse clinical and cost outcomes than standard practices alone; and (b) to contrast models to unearth elements of context which would otherwise remain hidden from view.

Lessons from FRONTIER: For the FRONTIER project, the overburdened traditional model of GP-led MSK care delivery made the inclusion of contrastive ideas in theorising valuable. The realist evaluation was used to understand how different GP practices employ physiotherapists differently, in order to tease out the causal contribution of different facets of the FCPP model. One contrastive theory related to the upskilling and deskilling of GPs when physiotherapists are included in primary care. We theorized that the presence of physiotherapists in primary care unburdens physicians by attending to all the patients with MSK complaints but results in a de-skilling effect for GPs in relation to their MSK expertise. To contrast this, we theorized the opposite effect: that physiotherapists working in primary care help GPs upskill in relation to

For peer review only - http://bmjopen.bmj.com/site/about/guidelines.xhtml

MSK issues by exposure to specialized knowledge, skills and innovations that the physiotherapist brings to the surgery. These theories were then tested through data collection.

4. Envision broad system impacts beyond the immediate implementation setting

New models of service delivery can mean moving resources from one part of a system to another (e.g. moving staff from secondary to primary care). This movement of resources may relieve problems in the destination area but create new staff challenges in the areas where resources originated. Such re-organization efforts require theorizing the broader impacts as well as the longer-term ripple effects. It may be difficult to capture evidence to test theories of broad and longer-term impact. However, such theorizing may still be useful for future research in the field, as well as on-going programme monitoring.

FRONTIER Experience: FCPP is a model that requires the movement of physiotherapists from secondary to primary healthcare settings. In some cases, this has resulted in the depletion in part of senior physiotherapists in secondary care settings who treat more complex MSK cases than in primary care. Attention needs to be paid to ensuring senior staff are retained at least, in part, in secondary care to manage complexity and also to support junior staff in secondary care settings.

5. Incorporate rapidly evolving service developments and context changes (e.g COVID-19) into the theory testing process in real-time.

Rapid shifts in service development may require realist evaluators to abandon theories developed at the outset of a study in favor of theories that become increasingly relevant over time. The abrupt rupture in the health service landscape brought on by the 2020 COVID-19

For peer review only - http://bmjopen.bmj.com/site/about/guidelines.xhtml

pandemic exemplifies the need to study causal impact of contemporary and emergent changes in real time [15]. For rapidly changing models of service delivery, the output of the realist evaluation needs to account for new and divergent models that have emerged out of necessity over the course of the research project.

Frontier Experience: The COVID-19 pandemic has dramatically impacted the FCPP service delivery model during the realist evaluation. It resulted in a UK wide lockdown initiated in March 2020, alongside advice to stop the delivery of all non-essential face-to-face health and social care [16]. For the physiotherapy profession, this meant an immediate and considerable change to physiotherapy provision with a rapid shift to remote consultations, for which the Chartered Society published delivery guidance. Although this service change was planned for future implementation (https://www.longtermplan.nhs.uk/online-version) the advent of the pandemic expedited its introduction and created little time for preparation or training. Whilst some in the field considered this a beneficial service development that saved time and was more convenient for many patients, others questioned whether patients considered it a 'valid' consultation and raised concerns regarding the greater potential for misdiagnosis and service inefficiencies.

There is a potential for the shift to online service provision to have long-term implications for physiotherapy practice and therefore FCPP implementation, although the extent of this impact remains uncertain. In addition to the implications for practice, the shift has had implications for the proposed FCP programme theories, as the theories to date have been generated on the basis of physical ('in house') co-location of FCPs in primary care.

For peer review only - http://bmjopen.bmj.com/site/about/guidelines.xhtml

Discussion

There is a need to maintain an iterative and adaptive position to theorizing in realist evaluation implementation as contexts change rapidly. It may feel necessary to abandon early theorizing if this becomes out of step with current developments in the service architecture. This is a normal process and it takes time to achieve clarity regarding important aspects of programmes for theory testing. The rapid evolution that new initiatives undergo can be as a result of overcoming implementation barriers created by new policy. In this regard, the foci of theory development and testing may shift over the duration of a realist investigation. This is advantageous as the outputs of such realist evaluations may yield important insights regarding programme success and failure that can be carried forward in future research and programme monitoring.

In addition, the creative thinking that occurs at the outset of a realist study can be beneficial to study programmes in evolution as it increases the agility that research teams need to adapt theory and research protocols. Inevitably, some theories will always be difficult to test due to a lack of available data. Nonetheless the development of such theories contributes to a cumulative body of work that lends itself to future study. Initial theorizing may also yield an over abundance of theories (e.g., n>30), requiring teams to consolidate and prioritize those theories to move forward for testing. An important lesson from the FRONTIER study is that the creative thinking conducted at the outset of the study was invaluable even if not all of it was taken forward. We found that the initial stage of our realist investigation was as much ‘theory development’ as it was ‘theory sensitization.’

For peer review only - http://bmjopen.bmj.com/site/about/guidelines.xhtml

As health systems evolve worldwide, it is necessary that methodologies such as Realist Evaluation be used and developed to capture the real-time changes and their causal impacts, to serve the needs of programme implementation. Through the reflections presented in this paper, we hope our demonstration of realist evaluation and the FRONTIER experience will help other teams improve the design of studies used to assess emerging and rapidly changing service delivery models.

Declarations

Ethics approval and consent to participate: IRAS261530

Consent for publication: not applicable

Availability of data and materials: not applicable

Competing interests: The authors declare no competing interests

Funding:

This study is funded by the National Institute for Health Research (NIHR) HS&DR HIHR programme (16/116/03)/name of part of the NIHR]. The views expressed are those of the authors and not necessarily those of the NIHR or the Department of Health and Social Care.

Authors' contributions: JJ wrote was responsible for submitting the manuscript. Co-authors HS, SH, RT, CL, NW provided multiple rounds of feedback. Authors MC, PK, DF, FC provided feedback on the final draft. All authors were involved in the design and analysis phases of the FRONTIER project.

For peer review only - http://bmjopen.bmj.com/site/about/guidelines.xhtml

Acknowledgements: not applicable

References

1. Pawson, R. and N. Tilley, *Realistic Evaluation*. 1997, London: Sage.
2. Jagosh, J., *Realist Synthesis for Public Health: Building an Ontologically Deep Understanding of How Programs Work, For Whom, and In Which Contexts.* Annual Review of Public Health, 2019. **40**(1): p. 361-372.
3. Rycroft-Malone, J., et al., *Realist synthesis: illustrating the method for implementation research.* Implement Sci, 2012. **7**.
4. Jagosh, J., *Retroductive theorizing in Pawson and Tilley's applied scientific realism.* Journal of Critical Realism, 2020. **19**(2): p. 121-130.
5. Manzano, A., *The craft of interviewing in realist evaluation.* Evaluation, 2016. **22**(3): p. 342-360.
6. Dalkin, S.M., et al., *What's in a mechanism? Development of a key concept in realist evaluation.* Implementation Science, 2015. **10**(1): p. 49.
7. Jagosh, J., et al., *A realist evaluation of community-based participatory research: partnership synergy, trust building and related ripple effects.* BMC Public Health, 2015. **15**(1): p. 725.
8. Greenhalgh, J. and A. Manzano, *Understanding 'context' in realist evaluation and synthesis.* International Journal of Social Research Methodology, 2021: p. 1-13.
9. Hunter, R., et al., *Realist review.* International Review of Sport and Exercise Psychology, 2022: p. 1-24.
10. *Elective Care High Impact Interventions: First Contact Practitioner for MSK Services*, N.E.a.N. Improvement, Editor. 2019.
11. Goodman C, D.S., Gordon AL, Dening T, Gage H, Meyer J, Schneider J, Bell B, Jordan J, Martin F, Iliffe S, Bowman C, Gladman JRF, Victor C, Mayrhofer A, Handley M & Zubair M., *Optimal NHS service delivery to care homes: a realist evaluation of the features and mechanisms that support effective working for the continuing care of older people in residential settings*. 2013.
12. Dr Recebba Randell, K., Mr David Wilkinson, Mr Gerard Perez, Mrs Jane Millar, Ms Julie Croft, Professor (Elizabeth) Andrea Nelson, Professor Alan Pearman, Professor Andrew Long, Professor David Jayne, Professor Dawn Dowding, Professor Joanne Greenhalgh, Professor Jon Hindmarsh, Professor Peter Gardner., *A realist process evaluation of robotic surgery: integration into routine practice and impacts on communication, collaboration and decision making*. 2013.
13. *First Contact Practitioners and Advanced Practitioners in Primary Care: (Musculoskeletal): A Roadmap to Practice*, H.E. England, Editor. 2019.
14. Pawson, R., *Causality for beginners.*, N.R.M. Festival, Editor. (2008).
15. Available from: https://www.csp.org.uk/system/files/publication_files/Remote%20consultations%20top%20tips%20v9.pdf.
16. Downey, A., *GPs urged to go digital to prevent spread of coronavirus*, in *Digital Health*. 2020.

For peer review only - http://bmjopen.bmj.com/site/about/guidelines.xhtml

For peer review only

For peer review only - http://bmjopen.bmj.com/site/about/guidelines.xhtml

BMJ Open

The benefits of realist evaluation for rapidly changing health service delivery models

Journal:	*BMJ Open*
Manuscript ID	bmjopen-2021-060347.R2
Article Type:	Communication
Date Submitted by the Author:	14-Jun-2022
Complete List of Authors:	Jagosh, Justin; University of the West of England<br>Stott, Hannah; University of the West of England<br>Halls, Serena; University of the West of England<br>Thomas, Rachel; University of the West of England<br>Liddiard, Cathy; University of the West of England<br>Cupples, Margaret; Queens University, Belfast, General Practice<br>Cramp, Fiona; The University of the West of England, Allied health professionals<br>Kersten, Paula; Canterbury Christ Church University, School of Health Sciences<br>Foster, Dave; University of the West of England<br>Walsh, Nicola; University of the West of England, Faculty of Health and Applied Sciences, Glenside Campus
**Primary Subject Heading**:	General practice / Family practice
Secondary Subject Heading:	General practice / Family practice
Keywords:	COVID-19, EDUCATION & TRAINING (see Medical Education & Training), PRIMARY CARE

SCHOLARONE™ Manuscripts

I, the Submitting Author has the right to grant and does grant on behalf of all authors of the Work (as defined in the below author licence), an exclusive licence and/or a non-exclusive licence for contributions from authors who are: i) UK Crown employees; ii) where BMJ has agreed a CC-BY licence shall apply, and/or iii) in accordance with the terms applicable for US Federal Government officers or employees acting as part of their official duties; on a worldwide, perpetual, irrevocable, royalty-free basis to BMJ Publishing Group Ltd ("BMJ") its licensees and where the relevant Journal is co-owned by BMJ to the co-owners of the Journal, to publish the Work in BMJ Open and any other BMJ products and to exploit all rights, as set out in our licence.

The Submitting Author accepts and understands that any supply made under these terms is made by BMJ to the Submitting Author unless you are acting as an employee on behalf of your employer or a postgraduate student of an affiliated institution which is paying any applicable article publishing charge ("APC") for Open Access articles. Where the Submitting Author wishes to make the Work available on an Open Access basis (and intends to pay the relevant APC), the terms of reuse of such Open Access shall be governed by a Creative Commons licence – details of these licences and which Creative Commons licence will apply to this Work are set out in our licence referred to above.

Other than as permitted in any relevant BMJ Author's Self Archiving Policies, I confirm this Work has not been accepted for publication elsewhere, is not being considered for publication elsewhere and does not duplicate material already published. I confirm all authors consent to publication of this Work and authorise the granting of this licence.

For peer review only

The benefits of realist evaluation for rapidly changing health service delivery models

Justin Jagosh
Centre for Advancement in Realist Evaluation and Synthesis (CARES)

Hannah Stott
Centre for Health and Clinical Research, University of the West of England (UWE), Bristol

Serena Halls
Centre for Health and Clinical Research, University of the West of England (UWE), Bristol

Rachel Thomas
Centre for Health and Clinical Research, University of the West of England (UWE), Bristol

Cathy Liddiard
Centre for Health and Clinical Research, University of the West of England (UWE), Bristol

Margaret E Cupples
Centre for Public Health Research, School of Medicine, Queen's University, Belfast

Fiona Cramp
Centre for Health and Clinical Research, University of the West of England (UWE), Bristol

Paula Kersten
Canterbury Christ Church University

Dave Foster
Patient Research Partner/Public contributor

Nicola Walsh
Centre for Health and Clinical Research, University of the West of England (UWE), Bristol

Correspondence to:
Justin Jagosh, Centre for Advancement in Realist Evaluation and Synthesis (CARES)
j.jagosh@realistmethodology-cares.org

For peer review only - http://bmjopen.bmj.com/site/about/guidelines.xhtml

Abstract

Realist evaluation is a methodology that addresses the questions: 'what works, for whom, in which circumstances, and how?' In this approach, programme theories are developed and tested against available evidence. However, when complex interventions are implemented in rapidly changing environments, there are many unpredictable forces that determine the programme's scope and architecture, as well as resultant outcome. These forces can be theorized, in real time, and included in realist evaluation outputs for current and future optimization of programmes. Reflecting on a realist evaluation of first-contact physiotherapy in primary care (the FRONTIER Study), five important considerations are described for improving the quality of realist evaluation outputs when studying rapidly changing health service delivery models. These are: (1) ensuring that initial programme theories are developed through creative thinking sessions, empirical and non-empirical literature, and stakeholder consultation; (2) testing the causal impact of formal and informal (e.g., emergent) components of service delivery models; (3) contrasting initial programme theories with rival theory statements; (4) envisioning broad system impacts beyond the immediate implementation setting; and (5) incorporating rapidly evolving service developments and context changes into the theory testing process in real-time (e.g., Additional Role Reimbursement Scheme, COVID-19). Through the reflections presented, the aim is to clarify the benefit of realist evaluation to assess emerging and rapidly changing service delivery models.

For peer review only - http://bmjopen.bmj.com/site/about/guidelines.xhtml

Introduction

Realist methodology is used for investigating how programmes work, for whom and in which circumstances [1, 2]. Although gaining widespread recognition for its value in assessing complexity across health and care sectors [3], guidance is sparse on using the realist approach to assess evolving models of health service delivery embedded in rapidly changing contexts. Shifting clinical need, heavy caseloads, competing health policies, and resource scarcity are a few reasons why health services may shift and evolve. Initiatives may also change due to practical necessities realized only during the implementation process. Complicating the evaluation of new efforts is the layering of interventions on existing services to address deficits and demands. As trends, needs and priorities shift, new models of service delivery may be terminated before substantial evidence of success or failure can accumulate. The resources of terminated interventions are often re-purposed for newer initiatives which are then launched again without a strong or insightful base of evidence. Realist evaluation is a suitable methodology to assess interventions under these conditions as the approach brings attention to the key hypothesized mechanisms of programmes and aspects of the context that matter, and such insights can be taken forward to other studies as programmes evolve and are re-purposed. In this way, realist evaluations cumulate efforts to not "re-invent the wheel" with every study, and to learn from the relevant programme theorizing that has come before [1]. This paper provides reflections on the FRONTIER study, which is a UK-based *National Institute for Health Research* (NIHR) funded study (https://fundingawards.nihr.ac.uk/award/16/116/03) examining first-contact physiotherapy (FCP). FCP introduces specialist, and in some cases, advanced practice physiotherapists into primary care settings to assess, diagnose, treat, and

For peer review only - http://bmjopen.bmj.com/site/about/guidelines.xhtml

manage patients presenting with musculoskeletal (MSK) disorders without the requirement for a prior general practitioner (GP) consultation [4]. FCP is a model developed as an alternative to traditional primary care service for MSK management in which patients are seen by a physiotherapist only after receiving a referral from a GP. In the new model, FCPs work at primary care sites, allowing patients direct access through self-referral or reception triage. The FCP embedding process is complex and impacts general practitioners, reception staff, patients, physiotherapists, and the wider health system. Whilst FCP services have been in existence over the last decade, their contribution to the primary care workforce has significantly increased more recently in response to the NHS Long Term Plan (https://www.longtermplan.nhs.uk/publication/nhs-long-term-plan/) and actioned through the Additional Roles Reimbursement Scheme (https://www.england.nhs.uk/wp-content/uploads/2019/12/network-contract-des-additional-roles-reimbursement-scheme-guidance-december2019.pdf). In England, the aim is for all adults to have access to a FCP by 2024 (https://www.england.nhs.uk/gp/expanding-our-workforce/first-contact-physiotherapists/).

Five important insights for conducting a realist evaluation in the context of rapidly evolving contexts are presented in Table 1. These considerations are explored further in the sections below, along with a brief description of the process of conducting a realist evaluation.

**Table 1: Five important considerations for conducting a realist evaluation for rapidly changing health service delivery models**
(1) Ensuring that initial programme theories are developed through creative thinking sessions, empirical and non-empirical literature, and stakeholder consultation.
(2) Analysing data to test the causal impact of formal and informal (e.g., emergent) components of service delivery models.

For peer review only - http://bmjopen.bmj.com/site/about/guidelines.xhtml

(3) Contrasting initial programme theories with rival theory statements (i.e., how the same resources can trigger very different responses and outcomes).

(4) Envisioning broad system impacts beyond the immediate implementation setting.

(5) Incorporating rapidly evolving service developments and context changes into the theory testing process in real-time (e.g., Additional Role Reimbursement Scheme, COVID-19).

Understanding realist evaluation

Realist evaluations of service delivery models typically involve the development of initial programme theories (IPTs) which are causal statements (e.g., 'if…then') hypothesizing how programme outcomes are manifested through programme mechanisms and corresponding contexts (see Table 2 for an example). Protocols are developed to collect and analyse data to test the IPTs. Deductive (theory-testing) as well as inductive (theory-gleaning) activities are used to build a proposed process to uncover underpinning explanatory mechanisms. This is known as *retroduction* (see definition in table 2) [5, 6]. Realist evaluation uses context-mechanism-outcome (CMO) configurations to achieve explanatory insights in theory development and data analysis. Programme mechanisms are understood to be underpinning generative forces that produce outcomes that activate in conducive contexts. Specifically, mechanisms are defined as the reasoning, response, or reaction by stakeholders (e.g., patients, staff) to programme resources. Programme resources may be formal or informal [2, 7]. Context may include aspects of causal impact that reside outside the scope of the programme's architecture [8, 9]. Outcomes can be quantitative or qualitative data typically seen as measurable impacts in behavioural, clinical, or system-level terms (see Table 2 for an example). Realist evaluations are often accompanied by a realist review (also known as realist synthesis)

For peer review only - http://bmjopen.bmj.com/site/about/guidelines.xhtml

[10], which is a literature-based realist analysis of programme theories related to the interventions under scrutiny.

Table 2: Definition and examples of terms used in realist evaluation

Context-Mechanism-Outcome Configuration: The central heuristic used in realist evaluation to understand what works, for whom, under which circumstances and how. Context is the backdrop of programmes whereas mechanism is how stakeholders respond to resources. Outcome is measurable impact at the behavioural, clinical or system level. For example, *Context: Physiotherapists often have more specialist knowledge regarding MSK conditions compared to general practitioners. Mechanism: First-point-of-contact physiotherapists are able to diagnose complicated MSK conditions in primary care and provide immediate access to tailored interventions for patients (resource) which reassures patients and physicians that patients are getting the timely MSK management they need (response). Outcome: Improved patient outcomes and satisfaction; increased staff satisfaction; fewer appointments required in onward referral; upskilling of GPs; fewer prescriptions.*

Initial Programme Theory: A hypothetical statement, often in the form of 'if…then,' that is developed at the start of a realist evaluation to explain how a programme or programme component works to produce outcomes. IPTs can take the form of rough (incomplete) CMO configurations. For example: *"If the primary care practice expects FCPs to allot a maximum of ten minutes to manage a MSK patient appointment in line with standard GP appointment length, and FCPs challenge this expectation based on a 20-minute appointment length which is standard to traditional physiotherapy, then GPs may perceive FCPs as being inefficient and may look to employ other practitioners in the future."*

Rival Theory: A hypothetical statement that shows how the same programme resources can lead to very different responses and outcomes. For example: *Rival Theory A – Physiotherapists working in primary care unburden GPs by attending to patients with MSK disorders. The reduced exposure to patients with MSK conditions results in GPs experiencing a de-skilling of MSK expertise. Rival Theory B - Physiotherapists working in primary care expose GPs to expert MSK management, resulting in GPs upskilling their MSK expertise.*

Contrastive Theory: A hypothetical statement that explains how a programme strategy works in comparison to a different programme strategy: For example: *If a FCP does not have injecting or prescribing qualifications then they will rely on traditional physiotherapy modalities for patient care (i.e., exercise, education, and lifestyle approaches). This contrasts with FCPs who do have said qualifications and may utilise injections and prescriptions more readily. The FCP who uses traditional modalities may provide improved patient outcomes over time over those who do not, due to the holistic approach inherent to those modalities.*

Programme Architecture: The complete set of strategies/components that comprise an intervention, both formally allocated and advised as well as informally assembled and adapted from local resources and deficits. For example, formal architecture of FCP includes banding, appointment length, reception staff triage training, and IT system integration. Informal architecture includes patient explanation about the FCP role, staff attitudes, and spontaneous interprofessional coordination efforts between GPs and physiotherapists. Some aspects of the informal architecture may become formalized over time. Realist evaluation is used to understand how a programme works by uncovering the mechanisms underpinning the programme's architecture.

Retroduction: A mode of inference that examines empirical outcomes in relation to the corresponding mechanisms of action that serve to produce them. For example: if a patient with an MSK disorder improves their condition by adhering to physiotherapy advice, it can be theorized that trust in the physiotherapist may increase patient motivation to uptake such advice. In this example, MSK disorder improvement is the outcome whereas trust and motivation are mechanisms. The theory is retroductive.

For peer review only - http://bmjopen.bmj.com/site/about/guidelines.xhtml

Important considerations for conducting a realist evaluation for rapidly changing health service delivery models

1. Ensure that initial programme theories are developed through a combination of creative thinking sessions, empirical and non-empirical literature, and stakeholder consultation

High-quality empirical literature is often sought to inform the development of IPTs at the outset of a realist evaluation. However, new service delivery models typically lack a trail of historical evidence regarding success and failure, and a lack of clarity on the programme's scope and architecture. These unknowns preclude easy identification of IPTs from pre-existing literature. For this reason, IPT development requires team-based creative thinking, consultation with key stakeholders at the early stages and retrieval of empirical as well as non-empirical literature. Such activities will bring initial programme theorizing in line with current developments and will likely produce relevant hypothetical insights about the mechanisms at play, which may be obscured in literature sources. During literature review, data sources should be expanded to include unpublished (grey) literature, policy documents, online blogs and forums, and from professional body documentation.

For the FRONTIER study, a six-month realist synthesis was conducted at the outset, to develop the IPTs and establish a suitable research scope to examine the most important facets of the emerging FCP service delivery model. The body of high-quality empirical literature on FCP was found to be limited and the few research papers retrieved did not describe clear programme theories that could be imported to the study. However, this literature was helpful to stimulate creative thinking and discussion within the team, which informed the development of relevant IPTs. Additional beneficial literature sources included policy documentation, online blogs and editorials in the UK-based physiotherapy professional body magazine 'Frontline'. In reading

For peer review only - http://bmjopen.bmj.com/site/about/guidelines.xhtml

policy documentation, the description and scope of FCP provided insight into the programme's formal, expected architecture. In the blog and editorial literature, physiotherapists candidly described experiences, successes and concerns while working in general practice. Such sources of literature provided greater insight into the informal, unexpected aspects of the programme and contemporary implementation issues.

2. Analyse data to test the causal impact of formal and informal (e.g., emergent) components of service delivery models

IPTs developed and tested in a realist evaluation can account for both the formal and informal architecture of programmes (see Table 2). This is particularly important for evolving models of health service delivery because, as formalized resources are shifted or removed, informal efforts are often needed to keep programmes afloat. Although policy documentation on service re-organization will describe formal architecture, implementation processes require additional undocumented efforts. A realist evaluation can capture these efforts, thus forming a comprehensive picture of how the programme works and how it evolves in real time.

For the FRONTIER study, the issue of pay scale, banding and associated skill-level exemplifies the importance of understanding formal and informal aspects of the programme. Documents from Health Education England (HEE) were scrutinized to understand the recommendations for how FCP roles should be implemented in primary care settings. HEE states that *"a First Contact Practitioner (FCP) is a diagnostic clinician working in Primary Care at the top of their clinical scope of practice at masters level Agenda for Change Band 7 or equivalent and above. This allows the FCP to be able to assess and manage undifferentiated and undiagnosed MSK presentations"* [11]. However, banding for FCP roles varies due to availability of appropriately

For peer review only - http://bmjopen.bmj.com/site/about/guidelines.xhtml

banded staff for work in general practice. Given this reality, it was observed that the architecture of FCP changes depending on the banding of the physiotherapist recruited to a primary care team. Physiotherapists at higher bands, while having greater experience and clinical capability, were able to offer prescriptions and injection which were frequently not within the scope of practice for lower banded physiotherapists working as FCPs. Through the realist evaluation it was theorized that, paradoxically, higher banded physios are potentially vulnerable to time pressures in primary care (i.e., reducing appointment length) leading to increased prescribing and injecting as opposed to engaging patients with core physiotherapy interventions. Alternatively, it was theorized that physiotherapists who were not qualified to prescribe and inject, were also not vulnerable to pressure to reduce appointment length. Traditional physiotherapy requires a longer appointment length than a GP appointment to engage patients with physical exercise, education, and communication. Longer appointment length also means increased time to establish trust and rapport with the patient. While formal guidance outlines general principles of FCP banding and pay structure, variation in banding at the local sites means that primary care managers are left on their own to try to understand and make decisions regarding FCP service architecture. Realist evaluation can be used to theorize these complexities and data collection can be conducted to better understand programme functioning given resource differences and limitations across local sites.

3. Contrasting initial programme theories with rival theory statements (i.e., how the same resources can trigger very different responses and outcomes)

Developing rival theories during the development of the IPTs can help to clarify aspects of the programme that are not well understood, especially in times of rapid context change. This is

For peer review only - http://bmjopen.bmj.com/site/about/guidelines.xhtml

because as contexts change, alternative theories may explain resultant shifts in the programme's architecture as well as successes and failures. Rival theories hypothesize how the same programme resources can lead to very different mechanism responses and outcomes, given general expectations of a new initiative. Similarly, contrastive theories can show how resources of a new initiative are expected to work differently when compared to older established practices (e.g., FCP versus GP-first models of care). Contrastive theories are also important to determine the clinical and cost effectiveness of new efforts. Two advantages in including realist contrastive and rival theories are (a) to help determine if new initiatives layered on existing services yield at least no worse clinical and cost outcomes than standard practices alone; and (b) to contrast models to unearth elements of context which would otherwise remain obscured.

In the FRONTIER study, the inclusion of rival and contrastive ideas in theorising was valuable as it helped to explore the overburdened traditional model of GP-led MSK care delivery. The realist evaluation was used to understand how different GP practices contract and employ physiotherapists, to tease out the causal contribution of different facets of the FCP model. One rival theory pertained to the upskilling and deskilling of GPs when physiotherapists are included in primary care. It was theorized that the presence of physiotherapists in primary care unburdens GPs by attending to many of the patients with MSK disorders but results in a de-skilling effect for GPs in relation to their MSK expertise. A rival theory was also explored: that physiotherapists working in primary care help GPs upskill in relation to MSK issues by exposure to specialized knowledge, skills, and innovations that the physiotherapist brings to the practice. These theories were then tested through data collection.

For peer review only - http://bmjopen.bmj.com/site/about/guidelines.xhtml

4. Envision broad system impacts beyond the immediate implementation setting

New models of service delivery can mean moving resources from one part of a system to another (e.g. moving staff from secondary to primary care). This movement of resources may relieve pressure in the destination area but create new staff challenges in the areas where resources originated. Such re-organization efforts require that realist evaluators theorize the broader impacts of the initiative, as well as the longer-term ripple effects. Although it may be difficult to capture evidence to test theories of broad and longer-term impact, such theorizing may still be useful for future research in the field, as well as for on-going programme monitoring.

The FRONTIER study team explored the idea that FCP is a model that attracts physiotherapists from secondary to primary care settings. It is suspected that some cases this migration has resulted in the depletion of senior physiotherapists in secondary care settings with consequent impact on supervision of junior staff and waiting list times. Such resource depletion may call into question the overall benefits of the FCP initiative across the pathway and create new pressures on physiotherapy services that modify the FCP model of care. The realist evaluation can theorize such impacts and either collect data to test such theories or produce recommendations for future research to investigate the wider impacts.

5. Incorporate rapidly evolving service developments and context changes into the theory testing process in real-time (e.g., Additional Role Reimbursement Scheme, COVID-19)

Rapid shifts in service development may require realist evaluators to abandon theories developed at the outset of a study in favour of theories that become increasingly relevant over time. The abrupt rupture in the health service landscape brought about by the 2020 COVID-19 pandemic exemplifies the need to study causal impact of contemporary and emergent changes in real time. For rapidly changing models of service delivery, the output of the realist evaluation

For peer review only - http://bmjopen.bmj.com/site/about/guidelines.xhtml

needs to account for new and divergent approaches that have emerged out of necessity over the course of the research.

The FRONTIER study collected data during the COVID-19 pandemic, which demonstrated how the pandemic dramatically impacted the FCP service delivery model. The UK- wide lockdown initiated in March 2020, alongside advice to stop the delivery of all non-essential face-to-face health and social care [12] created conditions for new programme theorizing in the realist evaluation. For the physiotherapy profession, the acceleration of on-line consultations meant an immediate and considerable change to physiotherapy provision with a rapid shift in the way consultations were conducted and managed. Although this service change was planned for future implementation (https://www.longtermplan.nhs.uk/online-version) the advent of the pandemic expedited its introduction and created little time for preparation or training. Realist evaluation can be used to theorize and investigate emerging developments by consulting expert practitioners and reviewing current policy documents. During FRONTIER data collection, it was found that whilst some FCP practitioners considered the transition to online service provision to be a beneficial service development that saved time and was more convenient for many patients, others questioned whether patients considered it a 'valid' consultation and raised concerns regarding the greater potential for misdiagnosis and service inefficiencies. These rival theories served to improve the vision of new service architecture when the current evidence-base was lacking. From a longitudinal perspective, there is a potential for the shift to online service provision to have long-term implications for physiotherapy practice and therefore FCP implementation, although the extent of this impact remains uncertain. In addition to the impact on practice, the shift has had implications for the proposed FCP

For peer review only - http://bmjopen.bmj.com/site/about/guidelines.xhtml

programme theories, as the original IPTs for the FRONTIER study were based on physical ('in house') co-location of FCPs with GPs in primary care. It is possible in a realist evaluation to construct new IPTs during data collection and test those theories with the remaining resources of the study.

Discussion

There is a need to maintain an iterative and adaptive position to theorizing in realist evaluation when contexts change rapidly, or the architecture of programming remains unknown. It may also feel necessary to abandon early theorizing if this becomes out of step with developments. These are normal processes in realist evaluation, and adequate time should be given to achieve clarity regarding important aspects of programmes for theory testing. The rapid evolution of new initiatives can be due to implementation barriers and resource constraints and the timeframe of a realist evaluation may overlap on such rapid change. In this regard, the foci of theory development and testing may shift over the duration of a realist investigation. This is advantageous as the outputs of such realist evaluations may yield important insights regarding programme success and failure that can be carried forward in future research and programme monitoring. In addition, the creative thinking that occurs at the outset of a realist study can be beneficial to study programmes in evolution, as this increases the agility that research teams need to adapt theory and research protocols. Inevitably, some theories will always be difficult to test due to a lack of available data. Nonetheless the development of such theories contributes to a cumulative body of work that lends itself to future study. Initial theorizing may also yield an over abundance of theories (e.g., n>30), requiring teams to consolidate and prioritize those theories for testing. An important lesson from the FRONTIER study is that the

For peer review only - http://bmjopen.bmj.com/site/about/guidelines.xhtml

creative thinking to develop IPTs conducted at the outset of the study was invaluable even if not all those theories were taken forward in data collection. It was found that the initial stage of the realist investigation was as much 'theory development' as it was 'theory sensitization.'

As health systems evolve worldwide, it is necessary that methodologies such as realist evaluation are used and developed to capture the real-time changes and corresponding causal impacts, to serve the needs of programme implementation. Through the reflections presented in this paper, we hope our demonstration of realist evaluation and experience in the FRONTIER study will help other teams improve the design of studies used to assess emerging and rapidly changing service delivery models.

For peer review only

Contributors: JJ wrote was responsible for submitting the manuscript. Co-authors HS, SH, RT, CL, MC, PK, FC, DF, and NW provided multiple rounds of feedback. All authors were involved in the design and analysis phases of the FRONTIER study.

Funding: The FRONTIER study is funded by the NIHR [HS&DR] (16/116/03)/HS&DR). The views expressed are those of the authors and not necessarily those of the NIHR or the Department of Health and Social Care. No additional funding was allocated for the development of this manuscript.

Competing interests: The authors declare no competing interests.

Open Access: I, the Submitting Author has the right to grant and does grant on behalf of all authors of the Work (as defined in the below author licence), an exclusive licence and/or a non-exclusive licence for contributions from authors who are: i) UK Crown employees; ii) where BMJ has agreed a CC-BY licence shall apply, and/or iii) in accordance with the terms applicable for US Federal Government officers or employees acting as part of their official duties; on a worldwide, perpetual, irrevocable, royalty-free basis to BMJ Publishing Group Ltd ("BMJ") its licensees and where the relevant Journal is co-owned by BMJ to the co-owners of the Journal, to publish the Work in BMJ Open and any other BMJ products and to exploit all rights, as set out in our licence.

For peer review only - http://bmjopen.bmj.com/site/about/guidelines.xhtml

The Submitting Author accepts and understands that any supply made under these terms is made by BMJ to the Submitting Author unless you are acting as an employee on behalf of your employer or a postgraduate student of an affiliated institution which is paying any applicable article publishing charge ("APC") for Open Access articles. Where the Submitting Author wishes to make the Work available on an Open Access basis (and intends to pay the relevant APC), the terms of reuse of such Open Access shall be governed by a Creative Commons licence – details of these licences and which Creative Commons licence will apply to this Work are set out in our licence referred to above.

References

1. Pawson, R. and N. Tilley, *Realistic Evaluation*. 1997, London: Sage.
2. Jagosh, J., *Realist Synthesis for Public Health: Building an Ontologically Deep Understanding of How Programs Work, For Whom, and In Which Contexts.* Annual Review of Public Health, 2019. **40**(1): p. 361-372.
3. Rycroft-Malone, J., et al., *Realist synthesis: illustrating the method for implementation research.* Implement Sci, 2012. **7**.
4. *Elective Care High Impact Interventions: First Contact Practitioner for MSK Services*, N.E.a.N. Improvement. NHS England, 2019.
5. Jagosh, J., *Retroductive theorizing in Pawson and Tilley's applied scientific realism.* Journal of Critical Realism, 2020. **19**(2): p. 121-130.
6. Manzano, A., *The craft of interviewing in realist evaluation.* Evaluation, 2016. **22**(3): p. 342-360.
7. Dalkin, SM., et al., *What's in a mechanism? Development of a key concept in realist evaluation.* Implementation Science, 2015. **10**(1): p. 49.
8. Jagosh, J., et al., *A realist evaluation of community-based participatory research: partnership synergy, trust building and related ripple effects.* BMC Public Health, 2015. **15**(1): p. 725.
9. Greenhalgh, J. and A. Manzano, *Understanding 'context' in realist evaluation and synthesis.* International Journal of Social Research Methodology, 2021: p. 1-13.
10. Hunter, R., Gorely, T, Beattie, M et al. *Realist review.* International Review of Sport and Exercise Psychology, 2022: p. 1-24.
11. *First Contact Practitioners and Advanced Practitioners in Primary Care: (Musculoskeletal): A Roadmap to Practice*, H.E. England, Editor. 2019.
12. Downey, A., *GPs urged to go digital to prevent spread of coronavirus*, in *Digital Health*. 2020.

For peer review only

For peer review only - http://bmjopen.bmj.com/site/about/guidelines.xhtml
